# The *Synthetic Microbiology Caucus*: from abstract ideas to turning microbes into cellular machines and back

**DOI:** 10.1111/1751-7915.13337

**Published:** 2018-11-20

**Authors:** Wei E. Huang, Pablo I. Nikel

**Affiliations:** ^1^ Department of Engineering Science University of Oxford Parks Road Oxford OX1 3PJ UK; ^2^ The Novo Nordisk Foundation Center for Biosustainability Technical University of Denmark 2800 Lyngby Denmark

The core tenet of synthetic biology is the application of engineering principles such as standardization, modularity and rational design to accelerate the *design‐build‐test* loop for reprogramming biological system by endowing them with novel tasks (Endy, 2005; de Lorenzo and Danchin, 2008; Church *et al*., 2014; Badenhorst and Bornscheuer, 2018; Kohman *et al*., 2018; de Lorenzo *et al*., 2018). Since its very inception as a field, synthetic biology has enabled researchers from different disciplines to extend and re‐think genetic manipulations as the rational design and engineering of cells. Against this background, (micro)organisms can be regarded as programmable cellular machines—which can be modified by manipulating the cells’ *software* (DNA/RNA), *hardware* (physical cell components), and the processes encompassing processing and regulation of nucleic acids and, even more importantly, metabolism (which, together, make up for the *operation system*; Rampley *et al*. 2017; Danchin, 2009). Moreover, in the context of contemporary synthetic biology practice, the *hardware* of cells can be purposefully defined by means of genome design and engineering, for which systems biology plays an important role. Synthetic biology and systems biology are viewed, in this sense, as two sides of the same coin. To fulfil this overarching engineering purpose, the ever‐expanding synthetic biology toolbox allows for the modification or tuning of gene network connectivities in a precise and in‐place manner. Decomposing, manipulating and re‐assembling the factors governing the *hardware*,* software* and *operation system* of the cell is crucial to understand its functioning *in toto*, e.g. for re‐factoring—the process by which a set of related genes are removed from their native regulatory context and placed under synthetic expression control.

As an enabling‐technology discipline, synthetic biology continues to provide sophisticated tools to precisely and rationally manipulate genetic programs, e.g. by adopting specific gene circuits, genetic toggle switches, amplifiers, sensors, (alternative) memories and oscillators. Yet, synthetic biology is still far from achieving its full potential in fields as diverse as healthcare, environmental protection, energy, agriculture, bio‐computing and efficient production of advanced chemicals and materials from renewable sources. Several challenges still remain along the road, e.g. undefined and incompatible bioparts, unpredictable gene circuit design, difficult‐to‐understand (let alone controlling) biological complexity and the appearance of phenotypic heterogeneities within isogenic populations (Kwok, 2010; Stephanopoulos, 2012; Vilanova *et al*., 2015; Wu *et al*., 2016). Furthermore, cells are living systems and, as such, they are constantly evolving (Bull and Barrick, 2017). Because of this unavoidable circumstance of every biological system, the introduction of designed gene circuits will likely interfere and cross‐talk with the gene networks defined by the indigenous genome. Such interactions between gene circuits and the host genome will influence the performance of gene expression and even crash with the functions of the implanted genetic devices. Moreover, since synthetic biology ultimately aims at programming cells that can execute the implanted functions in a predictable fashion, the adoption of specific, formatted hosts (biological *chassis*), endowed with different properties depending on the application envisioned, is still a matter in dispute (Calero and Nikel, 2018). Finally, several issues surrounding the use of genetically modified (micro)organisms, including intellectual property protection and open access to biological materials (Carbonell *et al*., 2016; Kahl *et al*., 2018), and the need of establishing efficient biocontainment strategies are also barriers in implementing synthetic biology, especially in practical (field) applications (Rampley *et al*. 2017; Whitford *et al*., 2018).

What is the state of affairs and the way ahead to tackle these challenges in the field? A combination of top‐down and bottom‐up approaches is used in synthetic biology, and the rational removal of genes and entire genomic regions has helped understanding the functions of genes and instruct the cell design in synthetic biology (Hutchison *et al*., 2016). The minimal genome project has helped to interrogate the essential genes that defines life, and the assembly of individual yeast chromosomes has been key to understand genetic regulations in a model microorganism. Looking at the *hardware* afresh has enabled to gain insight in structural organization of the bacterial cell, and a recent milestone in this sense has been the *in vitro* reconstitution of the (long neglected) bacterial cytoskeleton (Ramm and Schwille, 2018). Thus, understanding the wiring and functioning of the microbial cell at the structural level is a stepping stone in which synthetic biology can play a decisive role. In this sense, the metabolic *operation system* has been placed under the spotlight after years of undeserved ostracism (Nielsen, 2017), and the implementation of synthetic metabolisms has resulted in the design and construction of specialized cell factories that can utilize a range of substrates to produce complex, added‐value molecules (Smanski *et al*., 2016; Aslan *et al*., 2017; Nikel and de Lorenzo, 2018). These approaches rely on emerging analytical technologies for high‐resolution mapping of macromolecules and metabolites (Wang *et al*., 2016) supported by multi‐omic analyses and *big data* (Danchin *et al*., 2018). The use of xenonucleic acids is another fascinating aspect that continues to help expanding the repertoire of new‐to‐Nature cell functionalities (Schmidt *et al*., 2018); Furthermore, microbes usually (co‐)exist in communities, instead of isolated, single species. Some of the next major challenges would move from engineering single species towards the design of truly synthetic and programmable microbial communities, which would be able to perform more complex functionalities such as degradation of recalcitrant chemicals (Pelz *et al*., 1999)—taking full advantage of the division of metabolic labour (Brenner *et al*., 2008; Thommes *et al*., 2018), a feature that characterizes natural microbial communities (Nikel *et al*., 2014). Establishing integrated community systems with high‐level functionality would be the next step in this direction by understanding the factors governing assembly and maintenance of stable microbial consortia and synthetic microbiomes (Pham *et al*., 2017). Besides the clear applied angle of these approaches, synthetic biology now offers the tools and enabling technologies to explore both cellular and metabolic interactions of bacteria with other organisms beyond microbes, such as phages and potential hosts (both as symbionts and parasites). Understanding these processes from an engineering perspective will shed light on the bacterial lifestyle in different environments, the consequences of which will be relevant for fields as diverse as agriculture and medicine. Last, but certainly not least, most of the examples currently handy deal with the bacterial cell as a whole, but the adoption of cell‐free systems is also gaining momentum in the synthetic biology arena (Hodgman and Jewett, 2012).

Although the detailed enumeration of all the challenges that the synthetic biology community is facing would be burdensome, we believe that these aspects to constitute the next grand challenge for the field. An exciting brainstorming with Profs. Víctor de Lorenzo (CNB‐CSIC, Spain), Philippe Marlière (GénoPole, France) and Kenneth Timmis lead to the inception of the *Synthetic Biology Caucus*: a novel section of Microbial Biotechnology, which will provide an agora for sharing, documenting and discussing fresh ideas—from the entirely abstract ones to completely applied biotechnology designs—and to attract critical and constructive comments from the members of the synthetic biology community. Contributors to the section will communicate their thoughts in a dynamic format to identify key (either original or long standing) research questions that could prompt projects and experiments. The drafting of grant proposals represents a specific case in which many of the arguments that are elaborated or vented during the process have an intellectual value in themselves and would deserve to be shared and discussed in the community. The section will be run by Pablo I. Nikel (The Novo Nordisk Foundation Center for Biosustainability, Denmark) and Wei Huang (Oxford University, United Kingdom). Pablo's expertise is focused on metabolic engineering and synthetic metabolism in bacteria; Wei's competences are related to biosensors and bioenergy, and single cell omics. The editors will work together with the members of the community to move forward the concepts of synthetic biology and to promote delivery of the many promises that the field entails. Although we will invite potential contributors to submit their articles to this section, we strongly encourage all synthetic biologists from both fundamental and applied fields to contribute ideas, suggestions and arguments to the Caucus: this will be an exciting opportunity to foster open communication and scientific brainstorming with a wide reach out in the community!

## References

[mbt213337-bib-0001] Aslan, S. , Noor, E. , and Bar‐Even, A. (2017) Holistic bioengineering: rewiring central metabolism for enhanced bioproduction. Biochem J 474: 3935–3950. 29146872 10.1042/BCJ20170377 PMC5688466

[mbt213337-bib-0002] Badenhorst, C.P.S. , and Bornscheuer, U.T. (2018) Getting momentum: from biocatalysis to advanced synthetic biology. Trends Biochem Sci 43: 180–198. 29426712 10.1016/j.tibs.2018.01.003

[mbt213337-bib-0003] Brenner, K. , You, L. , and Arnold, F.H. (2008) Engineering microbial consortia: a new frontier in synthetic biology. Trends Biotechnol 26: 483–489. 18675483 10.1016/j.tibtech.2008.05.004

[mbt213337-bib-0004] Bull, J.J. , and Barrick, J.E. (2017) Arresting evolution. Trends Genet. 33: 910–920. 29029851 10.1016/j.tig.2017.09.008 PMC5777322

[mbt213337-bib-0005] Calero, P. , and Nikel, P.I. (2018) Chasing bacterial *chassis* for metabolic engineering: a perspective review from classical to non‐traditional microorganisms. Microb Biotechnol. 10.1111/1751-7915.13292 PMC6302729 29926529

[mbt213337-bib-0006] Carbonell, P. , Gök, A. , Shapira, P. , and Faulon, J.L. (2016) Mapping the patent landscape of synthetic biology for fine chemical production pathways. Microb Biotechnol 9: 687–695. 27489206 10.1111/1751-7915.12401 PMC4993189

[mbt213337-bib-0007] Church, G.M. , Elowitz, M.B. , Smolke, C.D. , Voigt, C.A. , and Weiss, R. (2014) Realizing the potential of synthetic biology. Nat Rev Mol Cell Biol 15: 289–294. 24622617 10.1038/nrm3767 PMC9645560

[mbt213337-bib-0008] Danchin, A. (2009) Bacteria as computers making computers. FEMS Microbiol Rev 33: 3–26. 19016882 10.1111/j.1574-6976.2008.00137.x PMC2704931

[mbt213337-bib-0009] Danchin, A. , Ouzounis, C. , Tokuyasu, T. , and Zucker, J.‐D. (2018) No wisdom in the crowd: genome annotation in the era of big data – current status and future prospects. Microb Biotechnol 11: 588–605. 29806194 10.1111/1751-7915.13284 PMC6011933

[mbt213337-bib-0010] Endy, D. (2005) Foundations for engineering biology. Nature 438: 449–453. 16306983 10.1038/nature04342

[mbt213337-bib-0011] Hodgman, C.E. , and Jewett, M.C. (2012) Cell‐free synthetic biology: thinking outside the cell. Metab Eng 14: 261–269. 21946161 10.1016/j.ymben.2011.09.002 PMC3322310

[mbt213337-bib-0012] Hutchison, C.A. , Chuang, R.Y. , Noskov, V.N. , Assad‐Garcia, N. , Deerinck, T.J. , Ellisman, M.H. , *et al* (2016) Design and synthesis of a minimal bacterial genome. Science 351: aad6253. 27013737 10.1126/science.aad6253

[mbt213337-bib-0013] Kahl, L. , Molloy, J. , Patron, N. , Matthewman, C. , Haseloff, J. , Grewal, D. , *et al* (2018) Opening options for material transfer. Nat Biotechnol 36: 923. 30307930 10.1038/nbt.4263 PMC6871013

[mbt213337-bib-0014] Kohman, R.E. , Kunjapur, A.M. , Hysolli, E. , Wang, Y. , and Church, G.M. (2018) From designing the molecules of life to designing life: future applications derived from advances in DNA technologies. Angew Chem Int Ed Engl 57: 4313–4328. 29316123 10.1002/anie.201707976

[mbt213337-bib-0015] Kwok, R. (2010) Five hard truths for synthetic biology. Nature 463: 288–290. 20090726 10.1038/463288a

[mbt213337-bib-0016] de Lorenzo, V. , and Danchin, A. (2008) Synthetic biology: discovering new worlds and new words. EMBO Rep 9: 822–827. 18724274 10.1038/embor.2008.159 PMC2529360

[mbt213337-bib-0017] de Lorenzo, V. , Prather, K.L. , Chen, G.Q. , O'Day, E. , von Kameke, C. , Oyarzún, D.A. , *et al* (2018) The power of synthetic biology for bioproduction, remediation and pollution control: the UN's Sustainable Development Goals will inevitably require the application of molecular biology and biotechnology on a global scale. EMBO Rep 19: e45658. 29581172 10.15252/embr.201745658 PMC5891403

[mbt213337-bib-0018] Nielsen, J. (2017) Systems biology of metabolism. Annu Rev Biochem 86: 245–275. 28301739 10.1146/annurev-biochem-061516-044757

[mbt213337-bib-0019] Nikel, P.I. , and de Lorenzo, V. (2018) *Pseudomonas putida* as a functional *chassis* for industrial biocatalysis: from native biochemistry to *trans*‐metabolism. Metab Eng DOI: 10.1016/j.ymben.2018.05.005 29758287

[mbt213337-bib-0020] Nikel, P.I. , Silva‐Rocha, R. , Benedetti, I. , and de Lorenzo, V. (2014) The private life of environmental bacteria: pollutant biodegradation at the single cell level. Environ Microbiol 16: 628–642. 24341371 10.1111/1462-2920.12360

[mbt213337-bib-0021] Pelz, O. , Tesar, M. , Wittich, R.M. , Moore, E.R. , Timmis, K.N. , and Abraham, W.R. (1999) Towards elucidation of microbial community metabolic pathways: unravelling the network of carbon sharing in a pollutant‐degrading bacterial consortium by immunocapture and isotopic ratio mass spectrometry. Environ Microbiol 1: 167–174. 11207732 10.1046/j.1462-2920.1999.00023.x

[mbt213337-bib-0022] Pham, H.L. , Ho, C.L. , Wong, A. , Lee, Y.S. , and Chang, M.W. (2017) Applying the design‐build‐test paradigm in microbiome engineering. Curr Opin Biotechnol 48: 85–93. 28419931 10.1016/j.copbio.2017.03.021

[mbt213337-bib-0023] Ramm, B. , and Schwille, P. (2018) *In vitro* reconstitution of the bacterial cytoskeleton: expected and unexpected new insights. Microb Biotechnol DOI: 10.1111/1751-7915.13336. PMC6302739 30411506

[mbt213337-bib-3000] Rampley, C.P.N. , Davison, P.A. , Qian, P. , Preston, G.M. , Hunter, C.N. , Thompson, P.I. , Wu, L.J. , Huang, W.E. (2017) Development of SimCells as a novel chassis for functional biosensors. Scientific Reports 7: 7261. 28775370 10.1038/s41598-017-07391-6 PMC5543166

[mbt213337-bib-0024] Schmidt, M. , Pei, L. , and Budisa, N. (2018) Xenobiology: state‐of‐the‐art, ethics, and philosophy of new‐to‐Nature organisms. Adv Biochem Eng Biotechnol 162: 301–315. 28567486 10.1007/10_2016_14

[mbt213337-bib-0025] Smanski, M.J. , Zhou, H. , Claesen, J. , Shen, B. , Fischbach, M.A. , and Voigt, C.A. (2016) Synthetic biology to access and expand nature's chemical diversity. Nat Rev Microbiol 14: 135–149. 26876034 10.1038/nrmicro.2015.24 PMC5048682

[mbt213337-bib-0026] Stephanopoulos, G. (2012) Synthetic biology and metabolic engineering. ACS Synth Biol 1: 514–525. 23656228 10.1021/sb300094q

[mbt213337-bib-0027] Thommes, M. , Wang, T. , Zhao, Q. , Paschalidis, I.C. , and Segrè, D. (2018) Designing metabolic division of labor in microbial communities. bioRxiv. 10.1101/442376 PMC6456671 30984871

[mbt213337-bib-0028] Vilanova, C. , Tanner, K. , Dorado‐Morales, P. , Villaescusa, P. , Chugani, D. , Frías, A. , *et al* (2015) Standards not that standard. J Biol Eng 9: 17. 26435739 10.1186/s13036-015-0017-9 PMC4591577

[mbt213337-bib-0029] Wang, Y. , Huang, W.E. , Cui, L. , and Wagner, M. (2016) Single cell stable isotope probing in microbiology using Raman microspectroscopy. Curr Opin Biotechnol 41: 34–42. 27149160 10.1016/j.copbio.2016.04.018

[mbt213337-bib-0030] Whitford, C.M. , Dymek, S. , Kerkhoff, D. , März, C. , Schmidt, O. , Edich, M. , *et al* (2018) Auxotrophy to Xeno‐DNA: an exploration of combinatorial mechanisms for a high‐fidelity biosafety system for synthetic biology applications. J Biol Eng 12: 13. 30123321 10.1186/s13036-018-0105-8 PMC6090650

[mbt213337-bib-0031] Wu, G. , Yan, Q. , Jones, J.A. , Tang, Y.J. , Fong, S.S. , and Koffas, M.A.G. (2016) Metabolic burden: cornerstones in synthetic biology and metabolic engineering applications. Trends Biotechnol 34: 652–664. 26996613 10.1016/j.tibtech.2016.02.010

